# Nrf2 and NF-κB and Their Concerted Modulation in Cancer Pathogenesis and Progression

**DOI:** 10.3390/cancers2020483

**Published:** 2010-04-13

**Authors:** Ilaria Bellezza, Anna Lisa Mierla, Alba Minelli

**Affiliations:** Dipartimento Medicina Sperimentale Scienze Biochimiche, Sezione Biochimica Cellulare, Università di Perugia, Via del giochetto, 06124 Perugia, Italy; E-Mails: aminelli@unipg.it (A.M.); annalisamierla@libero.it (A.L.M.)

**Keywords:** oxidative stress, chemoprevention, chemotherapy

## Abstract

Reactive oxygen species, produced by oxidative stress, are implicated in the initiation, promotion, and malignant conversion of carcinogenesis through activation/suppression of redox-sensitive transcription factors. NF-E2-related factor 2 (Nrf2) encodes for antioxidant and general cytoprotection genes, while NF-κB regulates the expression of pro-inflammatory genes. A variety of anti-inflammatory or anti-carcinogenic phyto-chemicals suppress NF-κB signalling and activate the Nrf2-ARE pathway. In this review we consider the role of Nrf2 and NF-κB in cancer pathogenesis and progression, focusing on their concerted modulation and potential cross-talk.

## 1. Introduction

Environmental toxicants and/or endogenous oxidants are responsible for chemical insults that represent one of the several causes for cancer pathogenesis. Indeed, most environmental carcinogens with a potential role in cancer initiation, promotion, and malignant conversion exert genotoxic and cytotoxic effects *via* a bio-activation process by cytochrome P450 (CYP), *i.e.*, large and diverse superfamily of hemeproteins representing phase I enzymes [1], and reactive oxygen species (ROS), produced by oxidative stress. Cumulative cancer risk increases with the fourth power of age and is associated either with an accumulation of DNA damage [2] or ROS, *i.e.*, the major source of endogenous DNA damage in aerobic organisms [3]. Many of the oxidatively-induced DNA lesions [4] are mutagenic and/or lethal, and may therefore be critical in carcinogenesis [5]. Due to several aging-associated alterations in the electron transport chain, the rate of ROS production increases with aging [6], thus resulting in a progressive increase in ROS and ROS-derived damage [7]. Many transcription factors, essential for gene expression/regulation as response to intracellular /environmental signals, are redox-sensitive proteins. In this review we will analyze the role of NF-E2-related factor 2 (Nrf2) and Nuclear factor (NF)-κB and their concerted modulation in cancer pathogenesis and progression.

**Scheme 1:**
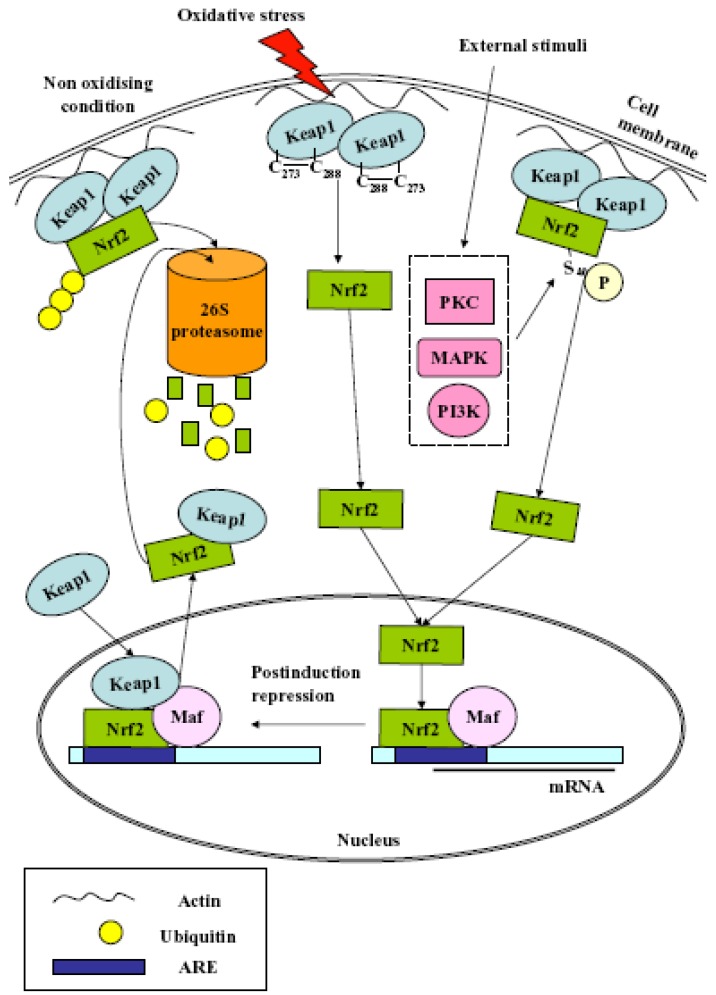
Proposed mechanisms for Nrf2 activation. Under normal homeostatic conditions, Nrf2 is retained in the cytoplasm *via* its interaction with Keap1 and degraded by proteasome. Oxidative stress causes disulfide bond formation between Cys273and 288 in Keap1 leading to Nrf2 release and nuclear translocation; External stimuli induce activation of protein kinases responsible for Nrf2 Ser40phosphorylation and Keap1 dissociation; Keap1 can undergo nuclear-cytoplasmic shuttling leading to disruption of the ARE/Nrf2 interaction.

## 2. Nrf2

Nrf2, first cloned and characterized by its ability to bind to the NF-E2/AP-1 repeat in the promoter of the β-globin gene [8], is ubiquitously expressed in many organs [9] as a transcriptional activator for phase II genes by binding to Antioxidant Responsive Element (ARE) sequences [10,11]. Nrf2 activation depends on Keap1 (Kelch ECH Associating Protein 1), a cytoskeleton protein capable of binding actin filaments and Nrf2, thus preventing its nuclear translocation and acting as transcriptional repressor during basal conditions. Nrf2, in its bound form, is targeted towards ubiquitinylation and subsequent proteasomal degradation in a Keap1-dependent fashion. The half-life of Nrf2 in cells is no more than 30 min [12] and the equilibrium between its synthesis and degradation maintains the basal expression of phase II enzymes. At oxidative conditions, Nrf2 is released from Keap1 repression, translocates to the nucleus, forms heterodimer with small Maf (musculoaponeurotic fibrosarcoma) proteins, recognizes and binds to a cis-acting ARE, and eventually recruits the whole transcription machinery including the RNA polymerase II to transcribe its target genes [13]. Keap1 [14] and Nrf2 are redox sensors while Keap1 is involved in post-induction repression of Nrf2 as well [14,15]. The concerted up regulation of ARE-driven genes enables adaptation to increased concentrations of ROS, reactive nitrogen species, and numerous electrophilic compounds. Five models, not necessarily mutually exclusive, since distinct mechanisms may be operative in the presence of different physiological conditions, have been proposed to explain Nrf2 nuclear accumulation and the accompanying ARE-driven gene induction [11] (Scheme 1):

(1) Nrf2 release from cytoplasmic anchoring: under normal homeostatic conditions Nrf2 is retained in the cytoplasm by its interaction with Keap1. During redox stress, Nrf2 is released from Keap1 and translocates from the cytoplasm to the nucleus. The release is due to several effectors, *i.e.*, modification of Cys residues in Keap1, Protein Kinase C (PKC)-mediated Nrf2 phosphorylation at Ser40, casein kinase 2 (CK2), extracellular signal-regulated kinase 2 (ERK2), ERK5, glycogen synthase kinase-3β (GSK-3β), c-Jun N-terminal kinase 1 (JNK1), PKR-like ER-localized eIF2α kinase (PERK) and phosphoinositide-3-kinase (PI3K);
(2) Nrf2 release from nuclear sequestration and recruitment to ARE enhancers: Keap1 can undergo nuclear-cytoplasmic shuttling through a potential nuclear export signal (NES). Upon stress stimulation, the nuclear protein prothymosin α binds the Kelch-repeat domain and liberates Nrf2, thus activating its target genes;
(3) Nrf2 protein stabilization: under normal homeostatic conditions, Nrf2 is continuously degraded by the 26S proteasome in a Keap1-dependent fashion. Keap1 also binds Cullin-3 (Cul3) to form a core E3 ubiquitin ligase complex. This complex targets Nrf2 for degradation in a redox-dependent fashion. Recently, the existence of two distinct binding sites in the Nrf2 domain for the Kelch-repeat domain has been proposed. It has been postulated that Keap1 immobilizes these ubiquitin acceptor sites by tethering Nrf2 across the two Kelch-repeat domains, bringing them into close proximity to Cul3–Rbx1 thereby facilitating ubiquitylation. Non-ubiquitylated Nrf2 remains bound to the Keap1–Cul3–Rbx1 complex until Keap1 regains its substrate adaptor activity. Newly translated Nrf2 protein would bypass the Keap1–Cul3–Rbx1 complex and accumulate rapidly in the nucleus;
(4) Antagonism of Nrf2 nuclear-cytoplasmic shuttling: under normal homeostatic conditions, Nrf2 nuclear abundance is restricted by the existence of a redox-dependent nuclear-cytoplasmic shuttling that, in turn, is controlled through the presence of a single nuclear localization sequence (NLS) and two NES motifs in the transcription factor. The NES contains an embedded Cysteine, and it has been proposed that modification of this residue by oxidants or electrophiles prevents recognition of the motif by exportin 1, thus leading to its nuclear accumulation;
(5) Nrf2 gene induction: the *Nrf2* promoter contains two ARE sequences and xenobiotic response elements (XREs). Multiple single nucleotide polymorphisms in the promoter of human *Nrf2* can regulate its expression.

Protein phosphorylation, as the major posttranslational mechanism in signalling processes, has a central role in regulating the Nrf2 liberation process, stability, and nuclear translocation [16]. Transducing oxidative stress signals to ARE- mediated gene expression involve mainly three pathways, *i.e.*, the PKC, the mitogen-activated protein kinase (MAPK) cascades, and the PI3K. Alternatively, whereas phosphorylation stabilises Keap1, dephosphorylation promotes rapid degradation of Keap1 and hence stabilization of Nrf2 [17]. Multiple transcriptional regulators, *i.e.*, p160 family coactivator, CREB-binding protein (CBP)/p300 [18,19], and corepressors such as silencing mediator of retinoid and thyroid receptors (SMRT) and histone deacetylase (HDAC)3, interact with Nrf2 and are present in Nrf2 nuclear complex [20,21]. Phosphorylation of coactivators and/or corepressor can affect activation/inactivation of Nrf2-directed gene expression [18,19,20,21]. Under unstressed condition, Nrf2 is constantly degraded *via* Keap1-mediated ubiquitination that is counter balanced by constitutive Nrf2 translocation. Under oxidative conditions, Keap1-mediated ubiquitination is impeded, while Nrf2 nuclear translocation is elevated [22], thus resulting in the expansion of the pool of free Nrf2 proteins. However, the nuclear influx of Nrf2 is still determined by the intensity of oxidative stress and Keap1 still modulates the redox-sensitivity of Nrf2 by controlling the availability of free Nrf2 proteins. 

## 3. NF-κB

**Scheme 2:**
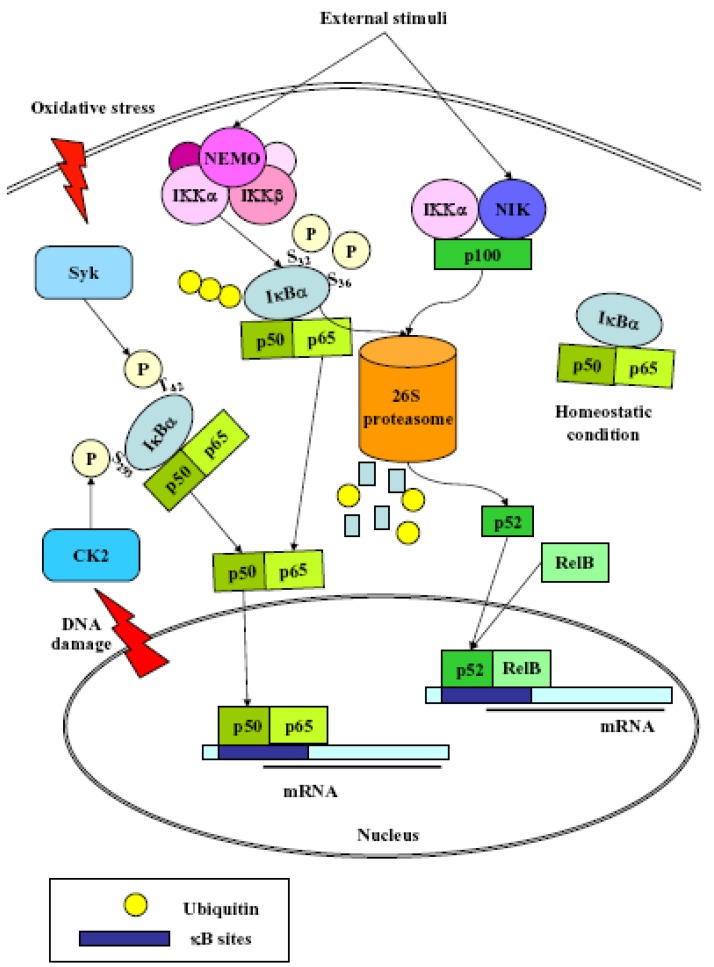
Proposed mechanisms for NF-κB activation. In basal conditions NF-κB is sequestered in the cytoplasm by IκBα. Upon stimulation, IκBα is rapidly phosphorylated at Ser32 and 36 by the IKK complex and degraded by the 26 S proteasome (classical pathway); DNA damage induces CK2 activation that leads to phosphorylation of IκBα at Ser293 and proteasome degradation (atypical pathway); oxidative stress induces Syk protein tyrosine kinase activation leading to IkBα Tyr42 phosphorylation and proteasome degradation (Oxidative stress-induced pathway). The released NF-κB dimer translocates to the nucleus and activates target genes by binding with high affinity to κB elements. External stimuli induce NIK and IKKα-dependent proteasomal processing of p100 into p52, which translocates to the nucleus and binds to DNA in association with RelB (alternative pathway).

NF-κB, defined as an inducible, widely expressed, pleiotropic transcription factor implicated in several physiological and pathological processes, represents a very efficient system for regulating gene expression [23]. The term NF-κB commonly refers to a p50-p65 heterodimer, which represents the major Rel/NF-κB complex in most cells. Four models have been proposed to explain NF-κB activation [24] (Scheme 2):

(1) Classical: induced by a variety of innate and adaptative immunity mediators, turned on within minutes. In basal conditions NF-κB is sequestered in the cytoplasm by inhibitor proteins, usually IκBα. Upon stimulation, IκBα is rapidly phosphorylated by the IkB kinase complex (IKK), which contains the catalytic subunits IKKα and IKKβ, the regulatory subunits NEMO and ELKS, and the heat shock protein Hsp90/Cdc37 chaperone complex [25]. Phosphorylated IκBα is substrate for ubiquitination and subsequent degradation by the 26 S proteasome [26]. The released NF-κB dimer then translocates to the nucleus and activates target genes by binding with high affinity to κB elements in their promoter;
(2) Alternative/NEMO-independent pathway: important for secondary lymphoid organ development, homeostasis and adaptive immunity, turned on in few hours. It involves the activation of NF-κB inducing kinase (NIK)- and IKKα-dependent proteasomal processing of p100 into p52, which binds DNA in association with its partners, *i.e.*, RelB [24]. NIK mediates p100 phosphorylation/ubiquitination in an IKKα-independent and IKKα-dependent manners. The phospho-p100, recognized and polyubiquitinated by the E3 ligase, is partially degraded by the proteasome to generate p52/RelB and p52/c-Rel or p52/p65 dimers. p52/RelB dimers move to the nucleus whereas p52/c-Rel and p52/p65 dimers, first captured by IκBs for their cytosolic retention, are activated through the classical pathway;
(3) atypical: independent from IKK, triggered by DNA damage such as UV [27] or doxorubicin [28], still requires the proteasome. UV radiation induces IκBα degradation *via* the proteasome, and the targeted serine residues are located within a C-terminal cluster, which is recognized by the p38-activated CK2;
(4) Oxidative stress-induced: relying on signal-induced phosphorylation events. NF-κB activation is achieved *via* IkBα tyrosine phosphorylation [29] by Syk protein tyrosine kinase [30]. H_2_O_2_ is the central second messenger to NF-κB activation [31] which involves several different mechanisms, since the redox-sensitive pathways triggering this activation are different depending on the cell-type considered [30]. NF-κB is also sensitive to oxidative modifications of Cys62 in p50, essential for DNA binding [32,33]. Its activation and nuclear translocation are stimulated by oxidizing conditions, while DNA binding is inhibited by the redox sensitive Cysteine residue [34,35].

Many upstream activators and regulators of IKK activity have already been identified, *i.e.*, NIK, mitogen-activated protein kinase/ERK kinase kinase (MEKK) 1, transforming growth factor β-activated kinase 1 (TAK1), PKCζ, MEKK2 and 3, and S6 kinase [35], but since diverse stimulants can all activate NF-κB, the range of kinases that activate IKK activity is expected to expand [36]. Besides phosphorylation and subsequent degradation of inhibitory molecules, protein kinases are required for optimal NF-κB activation by targeting functional domains of NF-κB proteins, such as GSK3β, TANK-binding kinase (TBK) 1, PKA, PKCζ. In most cases, these phosphorylations enhance p65 transactivation potential [36]. Transcriptional activity of NF-κB is also regulated by transcription coactivators and corepressors, *i.e.*, P300/CBP, P300/CBP-associated factor (p/CAF), and p160 proteins (SRC-1, SRC-2 and SRC-3) and SMRT, nuclear receptor corepressor (NCoR), HDAC1, HDAC2, and HDAC3, respectively [37]. 

## 4. Nrf2 and NF-κB Crosstalk

Several anti-inflammatory or anti-carcinogenic phytochemicals suppress NF-κB signalling and activate the Nrf2-ARE pathway [37,38], suggesting that the suppression of NF-κB signalling and the activation of the Nrf2-ARE pathway may crosstalk with each other. Depending on the level of ROS, different redox-sensitive transcription factors are activated and coordinate distinct biological responses. A low oxidative stress induces Nrf2, a transcription factor implicated in the transactivation of gene coding for antioxidant enzymes. An intermediate amount of ROS triggers an inflammatory response through the activation of NF-κB whereas a high level of oxidative stress induces perturbation of the mitochondrial permeability transition (PT) pore and disruption of the electron transfer, thereby resulting in apoptosis or necrosis [30,39].

Nrf2-deficient mice, subjected to a moderately severe head injury, show a greater cerebral NF-κB activation compared with their wild-type Nrf2 counterparts [40] while Nrf2 over expression suppresses NF-κB- DNA binding activity [41]. p65 protein levels, DNA-binding activity and promoter activity of NF-κB, increase in Nrf2 overespressing cells in the presence of curcumin, a polyphenolic compound that acts *via* Nrf2, suggesting that curcumin-activated Nrf2 modulates the expression and transactivation of NF-κB [42].

Cinnamaldehyde activates Nrf2 and the detoxifying phase II enzymes in endothelial cells [43]. This compound inhibits tumour necrosis factor (TNF) α-induced NF-κB activation *via* two distinct mechanisms that depend on pre-treatment time. In short term pre-treatments, cinnamaldehyde inhibits IκB-degradation whereas, over long term pre-treatments, the diterpene induces Nrf2-related genes, including hemeoxigenase 1 (HO-1). Changes in the thiol redox state of the cell, due to Nrf2 activation, may affect the post-translational modification of p65, including the phosphorylation of critical residues that contribute to its nuclear import [43]. Indeed, inhibition of NF-κB activation by phospholipid hydroperoxide glutathione peroxidase and 15-lipoxygenase is concomitant to up-regulation of HO-1, probably *via* Nrf2 activation [44]. Moreover, NF-κB -inhibited acute myeloid leukaemia cells do not undergo TNF-induced apoptosis and HO-1 is responsible for the resistance. Therefore, activation of Nrf2 by TNF under NF-κB -inhibited conditions is responsible of inhibition of apoptosis [45].

**Scheme 3:**
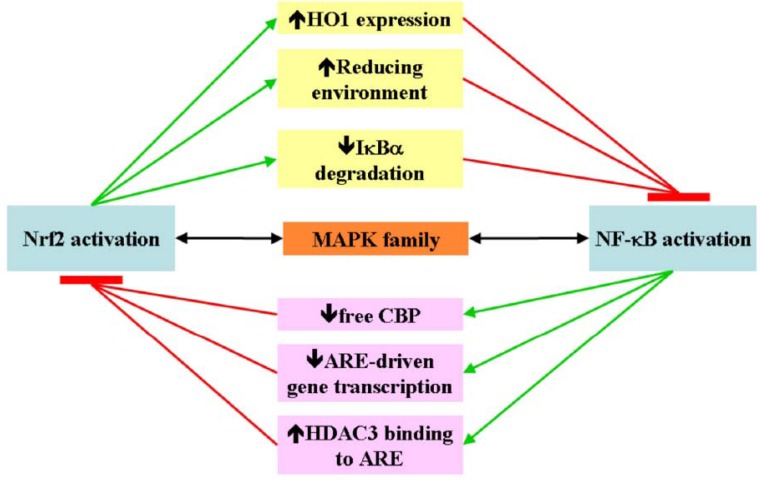
Proposed cross-talk between Nrf2 and NF-κB. Nrf-2 activation induces intracellular events that concur to NF-κB suppression and vice versa; MAPK family contributes to the concerted modulation of Nrf2 and NF-κB Green lines: induced events; red lines: resulting inhibitory events; black lines: concerted modulation.

NF-κB p65 subunit represses the Nrf2-ARE pathway at transcriptional level [20]. In cells where NF-κB and Nrf2 are simultaneously activated, p65 unidirectionally antagonizes the transcriptional activity of Nrf2 whereas in p65-overexpressing cells, the ARE-dependent expression of HO-1 is strongly suppressed. Nevertheless, p65 also inhibits the ARE-driven gene transcription independently from its own transcriptional activity. Indeed, p65 selectively deprives CBP from Nrf2 by competitive interaction that results in the Nrf2 inactivation which, in turn, depends on PKA catalytic subunit-mediated phosphorylation of p65 at Ser276. Moreover, p65 promotes recruitment of HDAC3, the corepressor, to ARE by facilitating the interaction of HDAC3 with either CBP or MafK, leading to local histone hypoacetylation. Collectively, the findings strongly support the participation of NF-κB p65 in the negative regulation of Nrf2-ARE signalling, providing a new insight into a possible role of NF-κB in suppressing the expression of anti-inflammatory or anti-tumour genes [20]. Epidemiological and clinical research corroborates an increased risk of certain cancers in the setting of chronic inflammation. Multiple sequence alignment of Nrf2 and NF-κB genes in five mammalian species (human, chimpanzee, dog, mouse and rat) has led to constructing a canonical regulatory network, involving several members of the MAPK family, for the concerted modulation of Nrf2 and NF-κB genes in inflammation/carcinogenesis [46] (Scheme 3). 

## 5. Nrf2 and NF-κB in Cancer Pathogenesis

Overall mortality rates from most cancers have not declined significantly in the past 30 years, thus preventive measures remain an integral part of the battle against cancer. One of the several causes for pathogenesis of cancer is the chemical insult caused by environmental toxicants and endogenous oxidants. Most environmental carcinogens exert their genotoxic /cytotoxic effects *via* a bio-activation process into electrophilic species by phase I enzymes, typically cytochrome P450s. These reactive species are often detoxified by phase II enzymes, *i.e.*, glutathione S-transferases (GSTs), UDP-glucuronosyl transferases (UGTs), sulfotransferases and NAD(P)H:quinone oxidoreductase 1 (NQO1), that conjugate the hydrophobic intermediates to a water-soluble group allowing the subsequent excretion. Under physiological conditions, these enzymes are constitutively expressed at low levels, but their expression levels can be greatly enhanced by a variety of chemical compounds including isothiocyanates, *i.e.*, sulforaphane, dithiolthiones, *i.e.*, oltipraz, diketopiperazine, *i.e.*, cyclo(His-Pro). Some of these compounds are effective anticarcinogens in animal models and are currently undergoing clinical evaluations as cancer chemopreventive agents. Protective response is mediated through the activation of the Nrf2 signalling pathway. Thus, Nrf2 and interacting factors are important targets either for drug discovery or elucidation of mechanisms of cytoprotective genes expression [1,47]. Since its discovery, Nrf2 has been viewed as a “good” protein that protects humans from genotoxic damage caused by carcinogens. Several *in vivo* studies with Nrf2-null mice confirmed the pivotal role of Nrf2 in cancer protection. Nrf2-null mice have reduced basal/ induced levels of phase II genes, *i.e.*, GST, NQO1, Glutamate–Cysteine ligase (GCL), and while displaying increased sensitivity to chemical toxicants and carcinogens, are refractory to the protective actions of chemopreventive compounds [1,14]. The induction of NF-κB pathways, generally associated with proliferation and resistance to apoptosis, contributes to tumourigenesis by transactivating several classes of target genes that have inflammatory, immunoregulatory, anti-apoptotic, and cell cycle regulatory functions. Several NF-κB-regulated genes, such as inducible nitric oxide synthase (iNOS) and cyclooxgenase-2 (COX-2), are characterised by pro-inflammatory properties thus connected to tumour promotion [48,49,50]. Based on the role of NF-κB in carcinogenic processes, it is plausible that this transcription factor can be the prime molecular target of chemopreventive drugs. 

On the other hand, DNA damage, oncogene activation and cellular stress induce DNA-binding and transcriptional activity of NF-κB [51], both required for p53-dependent apoptosis [52]. This surprising and controversial result implies that, in addition to functioning as a tumour promoter, NF-κB can potentially contribute to tumour suppression. p53 induces a switch from p52-Bcl-3 activator complexes to p52–HDAC repressor complexes, which results in the inhibition of the cyclin D1 promoter [53]. The association of p65 with HDAC1 turns NF-κB into a repressor of gene expression. Thus, NF-κB can act as a facilitator of apoptosis by repressing antiapoptotic gene expression [54]. This mechanism of NF-κB regulation might also be used in other situations, in which the NF-κB response needs to be curtailed. Therefore, the recruitment of HDAC3 to ARE [20], besides negatively regulating Nrf2, also exerts a positive cooperation since the action of genes under the control of NF-κB is helped by the simultaneous inhibition of the expression of the ARE-responsive genes. 

In the early stages of cancer, NF-κB might be tumour-suppressing rather than tumour-promoting. However, as the potential cancer cells accumulate more mutations, a selective pressure leads to decrease/abolish the expression of tumour-suppressor genes, thus reversing the NF-κB role and the mechanisms controlling the tumourigenic functions of NF-κB. Under these conditions, the tumour-promoting activity is unleashed and NF-κB subunits are free to induce the expression of a wide range of genes that promote the development of malignant and metastatic tumors. This two-step mechanism for NF-κB function in cancer development is probably tumour and cell-type specific [55]. There are some evidences that deregulation of p100 processing is associated to the emergence of haematopoietic and solid tumours [24]. Rearrangements of the *nfkb2* gene encode abnormal proteins that, lacking of the IκB-like inhibitory properties, become mainly nuclear and lead to a strong production of p52, responsible for the oncogenic phenotype. It is possible that Bcl3, one of the main partners of p52, over expressed in human clinical breast tumour specimens, interacts with p52 to form an oncogenic DNA-binding complex p52/p52/Bcl3 and play a role in breast tumourigenesis [24]. Thus, there is a strong possibility that, in conjunction with other factors, abnormal or subverted activation of the alternative NF-κB pathway contributes to cellular transformation.

## 6. Nrf2 and NF-κB in Cancer Progression

NF-κB subunits directly affect the growth and survival of a malignant tumour. Thereby, being resistance to apoptosis [56] one of the principal characteristics acquired by tumour cells, NF-κB activity can confer resistance to cell death in many types of tumour [57]. In particular, NF-κB can inhibit apoptosis induced by many common chemotherapeutic drugs and ionizing radiation [57,58]. These treatments stimulate NF-κB activity, therefore, even in tumours that do not display intrinsically aberrant levels of NF-κB activity; the inhibition of this signalling pathway could enhance the effectiveness of cancer therapy. Furthermore, NF-κB can stimulate angiogenesis, metastasis, cellular proliferation and the expression of many genes associated with tumour growth and survival [59,60,61] and elevation of NF-κB activity is evident in a number of human malignancies. Activation of NF-κB upregulates cyclin D1 [61] thus promoting cell cycle transition and anti-apoptotic genes, *i.e.*, cIAP1, cIAP2, XIAP, Bcl-2 and Bcl-X_L_, providing tumour cells with survival advantages [57,61]. The tumour suppressor gene p53 might control the putative oncogenic property of the DNA-binding complex p52/p52/Bcl3. Indeed, wild type p53 can induce the association of p52 homodimers with HDAC1 and the downregulation of Bcl3 protein, thus preventing the accumulation of p52/p52/Bcl3. Conversely, some tumour-derived p53 mutants induce *nfkb2* gene expression, which results in up regulation of anti-apoptotic genes and chemoresistance [24].

The role(s) of the ARE-driven genes in cancer chemoprevention and resistance to chemotherapeutic drugs is still debated. Indeed, GSTs isoenzymes, induced in mice and rats by chemopreventive agents, are overexpressed in hepatic pre-neoplastic nodules as a result from Nrf2 activation. GSH synthesis system is controlled by Nrf2 and consistent with Nrf2 activation, abundant glutathione (GSH) levels in tumour cell lines with acquired resistance to chemotherapeutic drugs are observed. Furthermore, Nrf2 up regulation results in overexpression of various drug-metabolising enzymes and drug-efflux pumps [11]. Up regulation of GSH-dependent enzymes is due to constitutive activation of normal stress responses caused by somatic mutations in the Keap1–Nrf2 pathway [11]. Keap1 mutations/loss of heterozygosity lead to Keap1 inactivation/reduced expression, which up regulates Nrf2 protein level and trans-activation of its downstream genes [14]. Sixty five Japanese patients with lung cancer showed a high incidence of Keap1 somatic mutations [62] and in breast cancer a Keap1 (C23Y) mutation impaired its ability to repress Nrf2 [63]. Under a hypoxia/reoxygenation condition, which mimics tumour microenvironment, Keap1 expression is decreased whereas Nrf2 and peroxiredoxin-1 (Prx1) up regulated, resulting in removal of ROS and protection of cancer cells [64]. Interestingly, by hypermethylation of the Keap1 promoter, an epigenetic mechanism, Keap1 expression was reduced in lung cancer cell lines and tissues, compared to that in normal bronchial epithelial cell line [65]. Consistently, Nrf2 was over expressed at later stages of cancer in lung tissue [14,66] and up regulation of Nrf2 was detected in an arsenic transformed human keratinocyte cell line [67]. These results suggest that loss of function of Keap1 may result in prolonged activation of Nrf2 providing cancer cells with a growth advantage due to up regulation of Nrf2 downstream genes [14]. Nrf2 may be also responsible for chemoresistance [11]. Contradictory reports exist on ROS levels and antioxidant defences in prostate cancer [68], characterised by high activities of catalase and glutathione peroxidase and high levels of GSH and metallothionein [69]. ROS level in LNCaP cells is higher than in PC3 cells, while GSH levels and GSH metabolizing enzyme activities are significantly higher in PC3 than in LNCaP cells [70] and in primary prostate tumors in mouse there is an increased oxidative damage [71,72,73]. In contrast, several classes of GSTs are downregulated with tumour progression [74] and Nrf2 is downregulated in human prostate cancer [75]. These findings suggest that maintaining normal Nrf2 expression and activity may be important in chemopreventative strategies for prostate cancer. Increased ROS generation in prostate cancer cells, *i.e.*, androgen-insensitive PC3, DU145, and androgen-sensitive LNCaP, may also be critical for migratory/invasiveness phenotypes [76].

## 7. Conclusions

Since its discovery, Nrf2 has been considered as playing a positive/protective role in cancer and in stress-related diseases. In healthy cells and tissues, Nrf2 protects from genotoxic damage caused by carcinogens and exerts a pivotal role in cancer protection by up regulating defensive genes. In cancerous cells and tissues, a completely different scenario can be found. Indeed, Nrf2 induction can provide cancer cells with growth advantage thereby causing resistance to chemotherapies. 

On the other hand, NF-κB has been strongly implicated in tumour promotion since it induces proliferation and resistance to apoptosis. However, in the early stages of cancer, NF-κB can potentially contribute to tumour suppression since DNA damage, oncogene activation and cellular stress induce DNA-binding and transcriptional activity of NF-κB, which is required for p53-dependent apoptosis. The activation of NF-κB, in cancerous settings, confers resistance to apoptosis, stimulates angiogenesis, metastasis, cellular proliferation and the expression of many genes associated with tumour growth and survival. Therefore, the inhibition of this signalling pathway could enhance the effectiveness of cancer therapy. These new perspectives might have implications for future NF-κB-based therapies and Nrf2- focussed chemopreventive strategies, although the very complex cross-talk, probably tumour and cell-type specific, between these (and other) transcription factors, might strongly influence the outcome of the prophylactic/therapeutic interventions.
